# A perilous path: the inborn errors of sphingolipid metabolism

**DOI:** 10.1194/jlr.S091827

**Published:** 2019-01-25

**Authors:** Teresa M. Dunn, Cynthia J. Tifft, Richard L. Proia

**Affiliations:** Department of Biochemistry,* Uniformed Services University of the Health Sciences, Bethesda, MD, 20814; Office of the Clinical Director and Medical Genetics Branch† National Human Genome Research Institute, Bethesda, MD 20892; Genetics of Development and Disease Branch§ National Institute of Diabetes and Digestive and Kidney Diseases, National Institutes of Health, Bethesda, MD 20892

**Keywords:** glycosphingolipids, metabolic disease, ceramides, bioactive lipids, gangliosides, storage diseases, rare disease, sphingolipids, genetics

## Abstract

The sphingolipid (SL) metabolic pathway generates structurally diverse lipids that have roles as membrane constituents and as bioactive signaling molecules. The influence of the SL metabolic pathway in biology is pervasive; it exists in all mammalian cells and has roles in many cellular and physiological pathways. Human genetic diseases have long been recognized to be caused by mutations in the pathway, but until recently these mutational defects were only known to affect lysosomal SL degradation. Now, with a nearly complete delineation of the genes constituting the SL metabolic pathway, a growing number of additional genetic disorders caused by mutations in genes within other sectors of the pathway (de novo ceramide synthesis, glycosphingolipid synthesis, and nonlysosomal SL degradation) have been recognized. Although these inborn disorders of SL metabolism are clinically heterogeneous, some common pathogenic mechanisms, derived from the unique properties and functions of the SLs, underlie several of the diseases. These mechanisms include overaccumulation of toxic or bioactive lipids and the disruption of specific critical cellular and physiological processes. Many of these diseases also have commonalities in physiological systems affected, such as the nervous system and skin. While inborn disorders of SL metabolism are rare, gene variants in the pathway have been linked to increased susceptibility to Parkinson’s disease and childhood asthma, implying that the SL metabolic pathway may have a role in these disorders. A more complete understanding of the inborn errors of SL metabolism promises new insights into the convergence of their pathogenesis with those of common human diseases.

## Graphical Abstract

**Figure:**
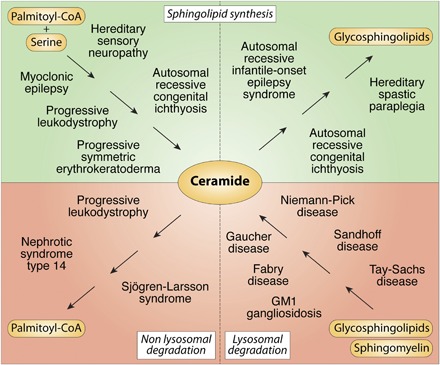

The metabolic pathway for sphingolipids (SLs), in various iterations, is a feature of all mammalian cell types (1). Its most basic function is to generate lipid building blocks for cell membranes, generally in the form of sphingomyelin and glycosphingolipids (GSLs). In this role, SLs impart essential physiochemical properties to membranes, act as receptors, and regulate the activity of membrane proteins (2). A second fundamental function of the SL pathway is to produce bioactive signaling molecules, such as sphingosine-1-phosphate (S1P), sphingosine, and ceramide, that interact with receptors or other targets to elicit biological responses (3, 4). Finally, sectors of the pathway have been specialized in a cell/tissue-specific manner to supply essential SLs with unique properties that are needed for key physiological functions [e.g., the production and transport of ultra-long chain ceramides for creating the skin permeability barrier (5)].

Mutational defects in the SL metabolic pathway, notably in lysosomal degradation, cause some of the oldest human metabolic diseases described. In recent years, the nearly complete inventory of the genes controlling the SL metabolic pathway, along with the advent of whole-exome sequencing analysis, has allowed the recognition, perhaps not surprisingly, of a growing number of additional disorders arising from genetic defects throughout the pathway (**Table 1**). Here, we document our understanding of the inborn errors of SL metabolism, their disease mechanisms, and how the increased understanding of these rare diseases is providing important insights into mechanisms underlying more common disorders.

**TABLE 1.** Inborn errors of sphingolipid metabolism

Disease (*GENE*)	Enzyme Defect/Lipid Defect	Major Systems Affected	References
Disorders of Ceramide Synthesis			
Hereditary sensory neuropathy type 1 (*SPTLC1* and *SPTLC2*)	Serine palmitoyl transferase/ deoxysphingolipids	• Nervous	(6, 10, 11)
Progressive symmetric erythrokeratoderma (*KDSR*)	3-Keto-dihydrosphingosine reductase/ceramide	• Skin	(12, 13)
		• Hematologic	
Autosomal recessive congenital ichthyosis (*CERS3*)	Ceramide synthase 3/ceramide	• Skin	(15, 16)
Myoclonic epilepsy (*CERS1*)	Ceramide synthase 1/ceramide	• Nervous	(17)
Progressive leukodystrophy (*DEGS1*)	Dihydroceramide desaturase/ceramide	• Nervous	(18)
Disorders of Glycosphingolipid Synthesis			
Autosomal recessive congenital ichthyosis (*UGCG*)	UDP-glucose ceramide glucosyltransferase/ glucosylceramide	• Skin	(25)
Autosomal recessive infantile-onset symptomatic epilepsy syndrome (*ST3GAL5)*	GM3 synthase/gangliosides	• Nervous	(26–28)
Hereditary spastic paraplegia (*B4GALNT1)*	GM2 synthase/gangliosides	• Nervous	(29, 30)
Disorders of Nonlysosomal Sphingolipid Degradation			
Progressive leukodystrophy (*ACER3*)	Alkaline ceramidase 3/ceramide	• Nervous	(33)
Nephrotic syndrome type 14 (*SGPL1*)	S1P lyase/S1P, sphingosine, ceramide	• Kidney	(35–37)
		• Skin	
		• Nervous	
		• Endocrine	
		• Immune	
Sjögren–Larsson syndrome (*ALDH3A2*)	Fatty aldehyde dehydrogenase/fatty aldehydes	• Nervous	(38)
		• Skin	
Disorders of Lysosomal Sphingolipid Degradation			
Farber lipogranulomatosis (*ASAH1*)	Acid ceramidase/ceramide	• Nervous	(39)
Fabry disease (*GLA*)	α-Galactosidase A/ globotriaosylceramide	• Kidney	(39)
		• Vascular	
		• Gastrointestinal	
Gaucher disease: type I, type II, type III, and perinatal lethal form (*GBA1*)	β Glucocerebrosidase, also known as β-glucosidase/ glucocerebroside, glucosylsphingosine	• Nervous	(39)
		• Skin	
		• Respiratory	
		• Hepatic	
		• Hematologic	
		• Skeletal	
GM1 gangliosidosis: type I, type II, and type III (*GLB1*)	β-Galactosidase/GM1 ganglioside	• Nervous	(39)
		• Skeletal	
GM2 gangliosidosis, Tay–Sachs disease (*HEXA*)	β-Hexosaminidase/GM2 ganglioside	• Nervous	(39)
GM2 gangliosidosis, Sandhoff disease (*HEXB*)	β-Hexosaminidase/GM2 ganglioside, GA2 glycolipid	• Nervous	(39)
GM2 gangliosidosis, GM2 activator deficiency (*GM2A*)	GM2 ganglioside activator/ GM2 ganglioside, glycosphingolipids	• Nervous	(39)
Globoid cell leukodystrophy, also known as Krabbe disease (*GALC*)	Galactosylceramidase/galactocerebroside, psychosine	• Nervous	(39)
Metachromatic leukodystrophy (*ARSA* and *PSAP*)	Arylsulfatase A and prosaposin/sulfatides	• Nervous	(39)
Niemann-Pick disease types A and B (*SMPD1*)	Sphingomyelin phosphodiesterase/ sphingomyelin	• Nervous	(39)
		• Hepatic	
		• Hematologic	
Disease Predisposition			
Childhood asthma (*ORMDL*3)	Serine palmitoyl transferase/ sphingolipid levels?	• Respiratory	(21, 23)
Ulcerative colitis (*ORMDL*3)	Serine palmitoyl transferase/ sphingolipid levels?	• Gastrointestinal	(22)
Parkinson’s disease (*GBA1*)	β Glucocerebrosidase, also known as β-glucosidase/ glucocerebroside, glucosylsphingosine	• Nervous	(45–47)

## DISORDERS OF SL SYNTHESIS: CERAMIDE

**Fig. 1.**
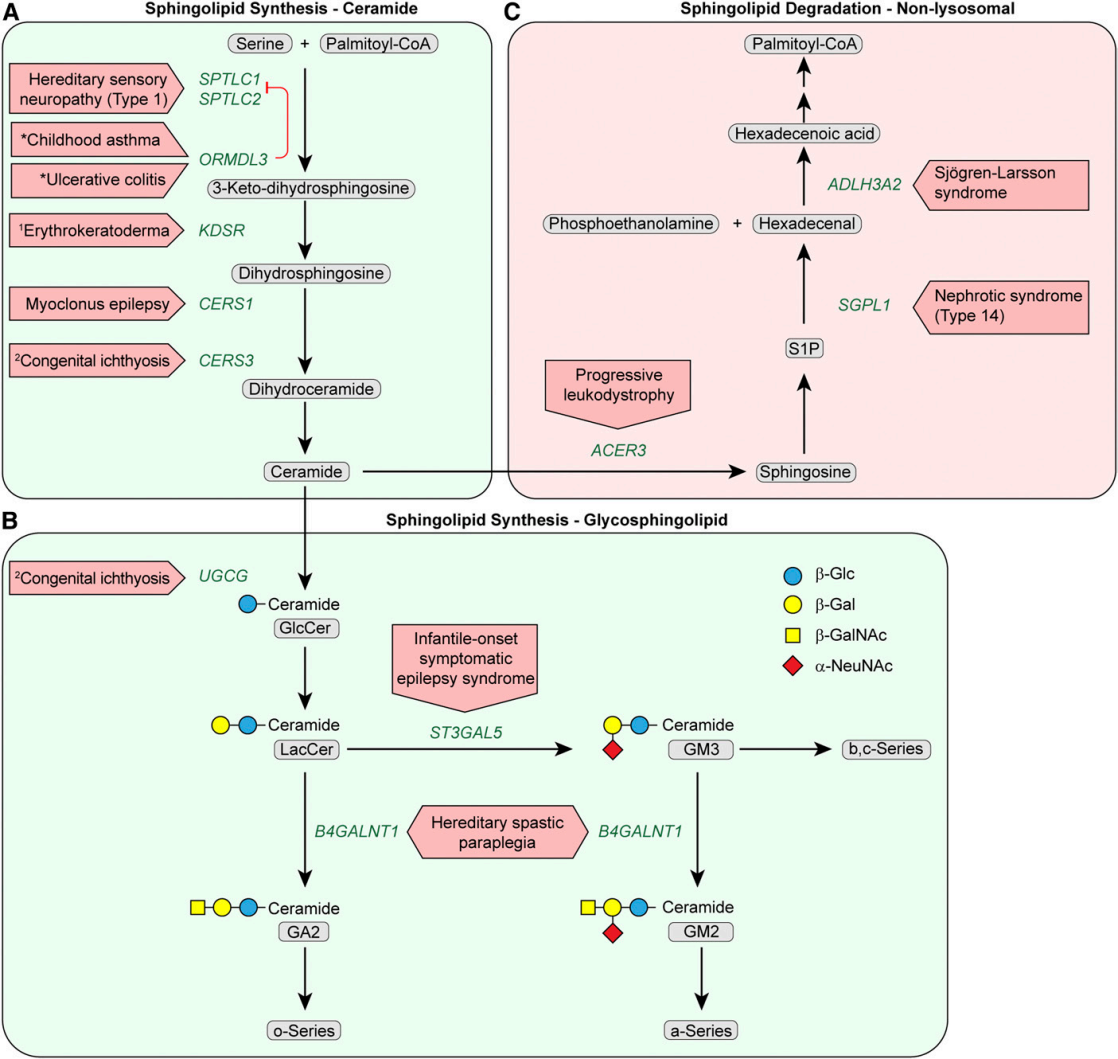
Disorders resulting from mutational defects in the SL synthesis pathway for ceramide and GSLs and the nonlysosomal SL degradation pathway. Sectors correspond to (A) de novo ceramide synthesis, (B) GSL synthesis, and (C) nonlysosomal SL degradation. Oligosaccharide structures are illustrated by colored symbols. The designations, o-Series, a-Series, b-Series and c-Series, refer to specific subgroups of GSLs and gangliosides (1, 24). Substrate names are presented in gray rounded boxes, genes are presented in green text, and disorders are presented in red boxed arrows. An asterisk identifies a disease predisposition associated with a gene variant. ^1^ Progressive symmetric erythrokeratoderma. ^2^ Autosomal recessive congenital ichthyosis.

**Fig. 2.**
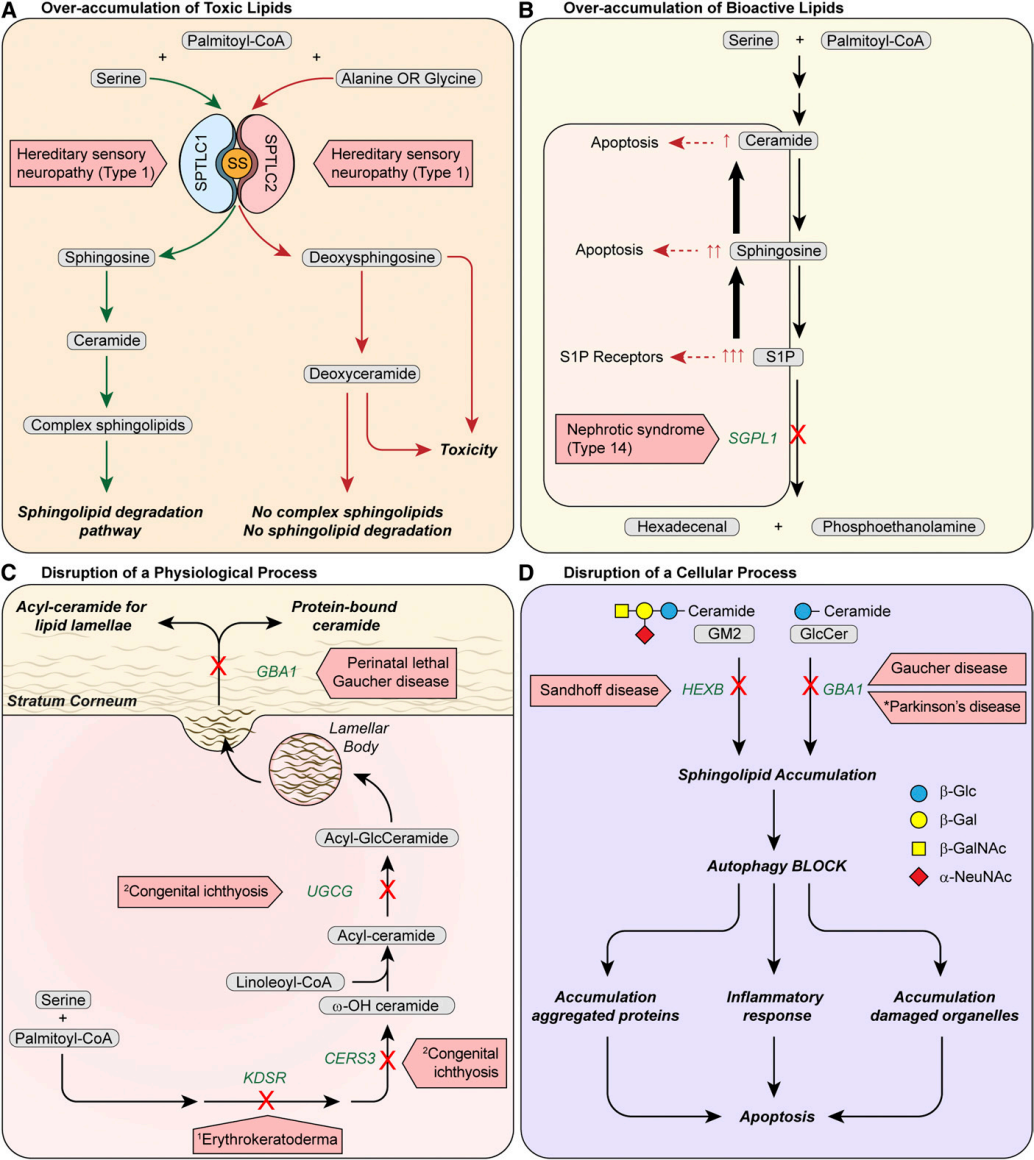
Mechanisms of pathogenesis of inborn errors of SL metabolism. A: Overaccumulation of toxic lipids. In HNAS1, mutations in either the SPTLC1 or SPTLC2 subunit of SPT increase utilization of alanine or glycine at the expense of serine for the increased production of deoxysphingosine. The deoxysphingosine can only proceed to deoxyceramide formation and cannot move farther down the pathway for complex sphingolipid synthesis or through the canonical degradation sphingolipid pathway. Accumulation of toxic deoxy-SLs result. SS: small subunit of SPT, SPTSSA. B: Overaccumulation of bioactive lipids. Mutations in S1P lyase cause nephrotic syndrome type 14 and block the only exit for sphingolipid substrate out of the SL metabolic pathway. As a consequence, S1P accumulates and some S1P is converted to sphingosine and ceramide, increasing levels of S1P, sphingosine, and ceramide. Each of these SLs is bioactive: excess S1P activates S1P receptors and sphingosine and ceramide trigger pathways causing apoptosis. In patients with SGPL1 deficiency, these SL alterations may contribute to the multisystemic manifestations in the disease (Table 1). The elevated circulating and tissue S1P levels may alter S1P receptor signaling to impair lymphocyte trafficking and cause immunodeficiency. Elevated levels of pro-apoptotic sphingosine and ceramide may contribute to neurologic symptoms. C: Disruption of a critical physiologic process. Establishment of the epidermal permeability barrier, which is essential for life, is critically dependent on the synthesis and metabolism of SLs. 3-Ketosphinganine reductase (*KDSR*) forms dihydrosphingosine and ceramide synthase 3 (*CERS3*), which is highly abundant in the epidermis, produces ceramide with very-long chain ω -hydroxy fatty acids, which are then acylated with linoleic acid to form acyl-ceramide. Glucosylation of acyl-ceramide by glucosylceramide transferase (*UGCG)* is believed to be required for the intracellular transport of lipids as lamellar bodies for secretion into the extracellular space at the stratum corneum. The secreted acyl-GlcCeramide is processed by glucocerebrosidase (*GBA1*) for proper formation of lipid lamellae and protein-bound ceramide in the stratum corneum to produce an intact permeability barrier. Biallelic mutations in each of the *KDSR*, *CERS3*, *UGCG*, or *GBA1* genes cause forms of epidermal permeability barrier abnormalities. D: Disruption of a critical cellular process. Lysosomal sphingolipid accumulation in Sandhoff *(HEXB*) and Gaucher (*GBA1*) diseases blocks autophagy, which is a critical self-degradative process for the removal of damaged organelles and aggregated proteins. An autophagy defect results in the cellular accumulation of damaged organelles and aggregated proteins and inflammation, which can lead to cellular dysfunction and apoptosis. Heterozygous mutations in *GBA1* increase the risk of Parkinson’s disease by possibly causing lysosome/autophagy dysfunction (47). Oligosaccharide structures are illustrated by colored symbols. Substrate names are presented in gray rounded boxes, genes are presented in green text, and disorders are presented in boxed red arrows. An asterisk identifies a disease predisposition associated with a gene variant. ^1^ Progressive symmetric erythrokeratoderma. ^2^ Autosomal recessive congenital ichthyosis.

Mutations in genes encoding enzymes and regulatory proteins controlling de novo synthesis of sphingoid bases (most often sphingosine) and ceramide produce a number of distinct human diseases (Table 1). For example, mutations in the SPTLC1 subunit of serine palmitoyl transferase (SPT) (**Fig. 1A**), the committed enzyme of de novo SL synthesis, has been reported to cause the rare autosomal dominant disorder hereditary sensory neuropathy type 1 (HSAN1) (6). Although it was initially thought that the mutations decreased SPT activity (7), the discovery of atypical deoxysphingosine in the sera of HSAN1 patients (8) led to the realization that the “gain-of-function” HSAN1 mutations confer promiscuity for amino acid substrate (9); that is, whereas wild-type SPT is highly selective for serine, the HSAN1 mutations permit relatively high utilization of alanine and glycine (thereby producing the deoxy-SLs implicated in neuronal damage; **Fig. 2A**). Several additional mutations in *SPTCL1* and in *SPTLC2* have been identified in HSAN1 patients, and all are associated with elevated deoxy-SLs (10, 11). Despite compelling evidence that the deoxy-SLs underlie the pathophysiology of HSAN1, the exact mechanism by which they damage neurons remains an area of active investigation.

The immediate product of SPT, 3-keto-dihydrosphingosine, is reduced by 3-ketosphinganine reductase to form dihydrosphingosine (Fig. 1A). Recessive mutations in *KDSR* cause inherited disorders of keratinization associated with thrombocytopenia (12, 13). Patients have skin lesions ranging in severity from those typical of the progressive symmetric erythrokeratoderma spectrum disorders to more serious harlequin ichthyosis-like lesions, thus placing *KDSR* in the growing family of genes associated with autosomal recessive congenital ichthyosis (14).

The identification of *KDSR* mutations in patients with disorders of keratinization is consistent with the well-appreciated role of ceramides as both critical components of the skin barrier and regulators of proliferation, differentiation, and apoptosis of keratinocytes. Indeed, mutations in several enzymes required for the synthesis of skin-specific acylceramides are also linked to autosomal recessive congenital ichthyosis. Included among these disease genes are: *CERS3* (15, 16) (Fig. 1A, 2C), encoding a ceramide synthase required for the synthesis of the epidermal very-long chain ceramides; and *UGCG* and *GBA1*, which are used for the synthesis and degradation of glucosylated acylceramides (Fig. 2C) (discussed below).

Biallelic mutations in another ceramide synthase (*CERS1*), which catalyzes the formation of C18 ceramides and is highly expressed in the brain, causes a novel progressive myoclonic epilepsy associated with neurodegeneration (17). In the final step of ceramide synthesis, a 4,5-trans-double bond is inserted into the dihydrosphingosine moiety of dihydroceramide by dihydroceramide desaturase, encoded by the *DEGS1* gene (Fig. 1A). Recently, a patient homozygous for a deleterious point mutation in the *DEGS1* gene was reported with progressive leukodystrophy and neurodegeneration (18). Defective ceramide synthesis in the nervous system may alter the production of critical gangliosides (discussed below).

The fact that perturbations in de novo ceramide synthesis underlie several human diseases points to the importance of tight regulation of the early steps in the pathway (19). As the committed and rate-limiting enzyme, proper control of SPT is likely pivotal to this regulation. The mammalian ORMDLs, a family of three highly related ER-associated proteins, have emerged as key regulators of SPT (20). Genome-wide association studies have provided evidence that altered *ORMDL3* expression is linked to risk for inflammatory disorders, including childhood asthma (21) and ulcerative colitis (22). For asthma risk, results conflict as to whether this is due to reduced SL levels resulting from enhanced ORMDL3-mediated inhibition of SPT (23). Understanding the relationship between *ORMDL3* expression, SPT activity, and inflammatory disease is an important goal, as is understanding the precise contributions of the ORMDLs to the overall regulation of SL homeostasis.

## DISORDERS OF SL SYNTHESIS: GSLS

GSLs are the largest subgroup within the SL family and are notable for their extremely diverse glycan head groups. They are the most prominent glycosylated lipids in mammalian cell membranes (24) and include the gangliosides (defined as GSLs carrying a sialic acid). The synthesis of the bulk of GSLs begins with the modification of ceramide with a β-linked glucose (Fig. 1B) by the enzyme UDP-glucose ceramide glucosyltransferase (also known as glucosylceramide transferase), encoded by the *UGCG* gene. After lactosylceramide is synthesized, GM3 synthase, encoded by the *ST3GAL5* gene, catalyzes the formation of GM3 ganglioside by the transfer of a sialic acid. Next, GM2 synthase, encoded by the *B4GALNT1* gene, transfers *N*-acetyl-β-galactosamine to lactosylceramide producing GA2 glycolipid and GM2 ganglioside, which then enables the synthesis of most complex gangliosides (Fig. 1B).

Human mutations that affect three steps in the GSL synthesis pathway have been described (Table 1). A recent report described an infant homozygous for a truncating mutation in the *UGCG* gene who was born with a collodion membrane and presented with a lethal form of ichthyosis (25), highlighting the importance of glucosylceramide transferase in the formation of the skin permeability barrier (Fig. 2C). GM3 synthase (*ST3GAL5)* deficiency, which was originally identified in Old Order Amish individuals, is responsible for an autosomal recessive infantile-onset symptomatic epilepsy syndrome associated with intractable seizures, developmental stagnation, extreme irritability, failure to thrive, cortical blindness, and cutaneous dyspigmentation (26–28). Genetic deficiency of GM2 synthase (*B4GALNT1)* has been described in individuals with a complex form of hereditary spastic paraplegia (29, 30). Prominent clinical features included progressive weakness and spasticity, as well as nonprogressive cognitive impairment.

Elimination of all complex gangliosides in KO mice produced lethal seizures in nearly 100% of the mice, illustrating the linkage between complex ganglioside deficiency and seizure activity (31). The mechanism that underlies the seizure activity in complex ganglioside deficiencies remains to be identified.

## DISORDERS OF SL DEGRADATION: NONLYSOSOMAL

After sphingosine generation by ceramidases, the terminal steps of SL degradation take place. First, S1P is formed via phosphorylation of sphingosine by sphingosine kinases. Next, the irreversible cleavage of S1P by S1P lyase (the *SGPL1* gene product) yields the nonSL substrates hexadecenal and phosphoethanolamine. For transfer to the glycerophospholipid pathway, hexadecenal is oxidized to hexadecenoic acid by the fatty aldehyde dehydrogenase encoded by the *ALDH3A2* gene. After CoA addition, hexadecenoyl-CoA is saturated to palmitoyl-CoA (Fig. 1).

Point mutations in the *ACER3* gene, which encodes a Golgi and ER-localized alkaline ceramidase with specificity for unsaturated long chain ceramides (32), result in a loss of enzyme activity and have been reported to cause early childhood-onset progressive leukodystrophy with developmental regression and peripheral neuropathy (Table 1) (33). *Acer3* KO mice exhibit a late-onset neurogenerative phenotype with elevated ceramides and other SLs in the brain, but no evidence of myelination defects (34). While these findings indicate that *ACER3* is critical for SL homeostasis in the brain, the mechanism underlying the neurodegeneration that occurs is not known.

Inactivating mutations in the *SGPL1* gene cause a recently recognized syndrome of steroid-resistant nephrotic pathologies associated with facultative ichthyosis, adrenal insufficiency, immunodeficiency, and neurological defects called nephrotic syndrome type 14 (35–37). Significantly, *Sgpl1* KO mice recapitulated many features observed in these patients. The pathogenesis of this disease may result from the overaccumulation of potent bioactive signaling lipids upstream of the block caused by the absence of S1P lyase (Fig. 2B).

Sjögren-Larsson syndrome is caused by mutational inactivation of the fatty aldehyde dehydrogenase *ALDH3A2*, which is required to transfer SL-derived substrates to the glycerol phospholipid pathway (38). The syndrome is characterized by ichthyosis, mental retardation, spastic paraparesis, macular dystrophy, and leukoencephalopathy. Accumulation of reactive fatty aldehydes that damage critical proteins in the skin and nervous system has been postulated to underlie the pathogenesis in the syndrome (38).

## DISORDERS OF SL DEGRADATION: LYSOSOMAL

**Fig. 3.**
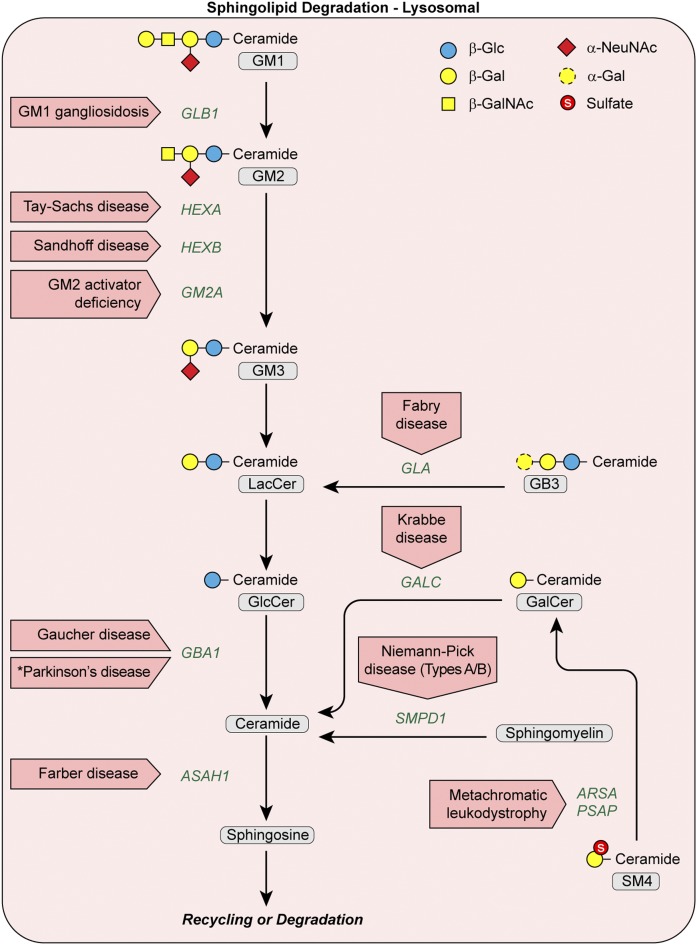
Disorders resulting from mutational defects in the lysosomal SL degradation pathway. Oligosaccharide structures are illustrated by colored symbols. Substrate names are presented in gray rounded boxes, genes are presented in green text, and disorders are presented in boxed red arrows. An asterisk identifies a disease predisposition associated with a gene variant.

Plasma membrane SLs, sphingomyelin, and GSLs undergo stepwise enzymatic degradation in lysosomes, with the final step generating sphingosine (**Fig. 3**). Genetic defects blocking a particular enzymatic degradation step in this pathway result in the accumulation of the normally degraded SL substrate and give rise to lysosomal storage disorders (LSDs) (Table 1). These SL LSDs are by far the most well-studied inborn errors of SL metabolism and are discussed as a group to illustrate their key features (39).

Three of the most common SL LSDs are Fabry disease (up to 2.5 cases per 100,000 males), metachromatic leukodystrophy (up to 2.5 cases per 100,000 individuals), and Gaucher disease (up to 2 cases per 100,000 individuals) (39). These figures are underestimates, because patients with later onset or atypical disease presentations may go unrecognized for years following the onset of symptoms or may never receive a diagnosis. Particular SL LSDs have an increased incidence in specific ethnic groups due to a founder effect (e.g., Tay-Sachs disease in the Ashkenazi Jewish population or GM1 gangliosidosis in regions of Brazil) (39). The prevalence of some of these disorders (e.g., Gaucher and Fabry diseases) in the population, which is a function of incidence and mean survival, is increasing with the advent of new therapies. Central nervous system degeneration is the dominant clinical feature in most of the SL LSDs, making the group difficult to study and difficult to treat in human populations (40) (Table 1). Other manifestations may be the result of unique functions of the specific disease gene, such as in perinatal lethal Gaucher disease (*GBA1*), a very severe disorder associated with ichthyosis (Fig. 2C) (41).

SL LSDs are autosomal recessive disorders with the exception of Fabry disease, which has been shown to be hemizygous, with females developing symptoms approximately 10 years later than their affected brothers. Many SL LSDs represent a continuum of disease severity, as well as variable age at onset and progression, based on the amount of residual enzyme activity, which is in turn governed by the specific mutation(s). Impaired autophagy, a result of SL storage, has been implicated in the pathogenesis of SL LSDs, including Sandhoff and Gaucher diseases (Fig. 2D) (42–44). Further, mutations in genes causing SL LSDs may predispose individuals to more common disorders. Astute clinical observation (45) leading to confirmation by a multi-center international consortium has now established Gaucher (*GBA1*) carrier status as the most common risk factor for Parkinson’s disease (PD) and Lewy body dementia (46). Lysosome/autophagy dysfunction is further implicated as a link between LSDs and PD based on findings that heterozygous mutations in LSD-related genes are overrepresented in patients with sporadic PD, including in the SL degradative genes *GBA1*, *ASAH1*, *SMPD1*, and *GALC* (Fig. 3) (47, 48).

## SUMMARY

The inborn errors of SL metabolism now comprise disorders caused by mutations in more than 20 different genes in the SL pathway (Table 1). Although generally clinically heterogeneous, some common features emerge. Many of the lysosomal SL degradation diseases present as infantile or childhood neurodegenerative disorders with defects in autophagy, pointing to both the essential nature of lysosomal SL metabolism in the central nervous system and the existence of common pathogenic mechanisms. Many of the gene mutations in other sectors of the SL metabolic pathway also affect the nervous system, suggesting that they may alter the proper levels and complement of critical gangliosides on neuronal membranes. Other neurologic disorders may cause pathogenesis by the elevation of toxic or bioactive SL metabolites. Skin pathology is a manifestation of several of these disorders, demonstrating the central importance of the pathway in skin permeability barrier development and function.

Although the inborn errors of SL metabolism are rare diseases, a detailed understanding of their pathogenic mechanisms can illuminate our understanding of common diseases. Susceptibility to PD and childhood asthma have been linked to genetic variants of *GBA1* and *ORMDL3*, respectively, tying these disorders directly to the SL metabolic pathway (21, 45, 46). Although not directly genetically linked to SL metabolism, cancer and diabetes exhibit alterations in SL levels and signaling that are believed to contribute to their progression (49, 50).

The significant advances made in our understanding of the biochemistry and genetics of the SL metabolic pathway have enabled the identification of new human genetic disorders beyond those in lysosomal SL degradation. This more complete understanding of the entire group of inborn errors of SL metabolism is yielding novel insights into disease mechanisms that are relevant to the pathogenesis of common human diseases.

## Supplementary Material

Supplemental Data
