# Supplementary material for: A perilous path: the inborn errors of sphingolipid metabolism
Source: J Lipid Res. 2019 Jan 25;60(3):475–83. doi: 10.1194/jlr.S091827 (PMC6399501; doi:10.1194/jlr.S091827)
Supplement: Supplemental Data [file supp_60_3_475__index.html]

A Perilous Path: The Inborn Errors of Sphingolipid Metabolism — A perilous path: the inborn errors of sphingolipid metabolism — Supplemental Data 

# A perilous path: the inborn errors of sphingolipid metabolism

## Supplemental Data

- graphical abstract (.jpg, 1.5 MB) - graphical abstract
