# Supplementary figures and images for: A perilous path: the inborn errors of sphingolipid metabolism
Source: J Lipid Res. 2019 Jan 25;60(3):475–83. doi: 10.1194/jlr.S091827 (PMC6399501; doi:10.1194/jlr.S091827)

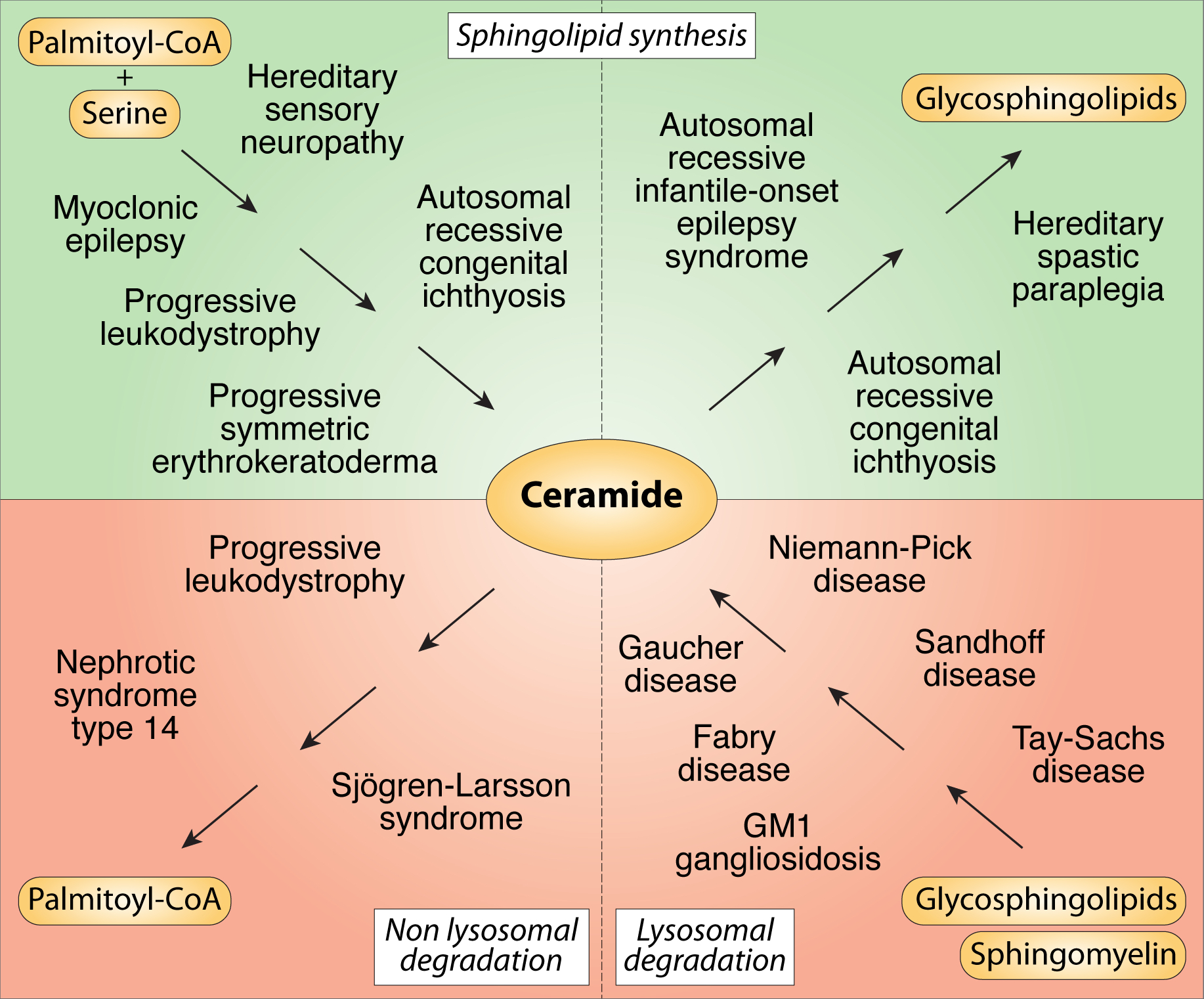

Supplement: Supplemental Data [file 10.1194_S091827_jlr.S091827-1.jpg]
